# Individual *Mycobacterium tuberculosis* universal stress protein homologues are dispensable *in vitro*

**DOI:** 10.1016/j.tube.2010.03.013

**Published:** 2010-07

**Authors:** S.M. Hingley-Wilson, K.E.A. Lougheed, K. Ferguson, S. Leiva, H.D. Williams

**Affiliations:** Department of Life Sciences, Faculty of Natural Sciences, Imperial College London, Sir Alexander Fleming Building, London SW7 2AZ, UK

**Keywords:** DosR, Hypoxia, Stationary phase, hspX

## Abstract

*Mycobacterium tuberculosis* has 10 universal stress proteins, whose function is unknown. However, proteomic and transcriptomic analyses have shown that a number of *usp* genes are significantly upregulated under hypoxic conditions and in response to nitric oxide and carbon monoxide, as well as during *M. tuberculosis* infection of macrophage cell lines. Six of these USPs are part of the DosR regulon and this, along with their expression pattern and the phenotypes of *usp* mutants in other bacterial species, suggests a potential role in the persistence and/or intracellular survival of *Mtb*. Knock-out mutants of individual *usp* genes encoding the USPs *Rv1996*, *Rv2005c*, *Rv2026c* and *Rv2028c* were generated and their growth and survival under hypoxic and other stress conditions examined. Although the majority of *usp* genes are highly induced in hypoxic conditions, mutation did not affect the long term survival of *Mtb* under these conditions, or in response to a range of stress conditions chosen to represent the environmental onslaughts experienced by the bacillus during an infection, nor during infection of mouse and human – derived macrophage cell lines. The possibility remains that these USPs are functionally redundant in *Mtb.*

## 1 Introduction

Over a third of the world’s population is thought to be latently or persistently infected with *Mycobacterium tuberculosis,* which represents a huge potential reservoir of infection.^1^ There is an urgent need to fully understand the host/pathogen interaction, in particular during this latent state, in order to develop improved vaccine, diagnostic and treatment strategies with which to curtail this disease. In addition, persistent bacilli may be responsible for the need for the long antibiotic treatment regimes required to treat TB effectively.^2^ Infection with *M. tuberculosis* (*Mtb*) generally occurs upon inhalation of the bacilli in airborne droplets. The bacilli are then taken up by alveolar macrophages and it is here that they survive and propagate where most other pathogens perish. Understanding the ability of the tubercle bacillus to persist during latent infection has been the object of much research in attempts to define both the initiating factor(s) and the bacterial genes required for non-replicating persistence. It is thought that *Mtb* will experience hypoxic conditions in the granulomas that form during chronic infection as they are avascular, inflammatory and necrotic, conditions which lead to low oxygen environment.^3–5^ *Mtb* can survive for long periods in a non-replicating anaerobic state.^6^ Wayne and co-workers developed a hypoxic model of non-replicating persistence,^7^ in which the shift-down from active replication to non-replicating persistence occurs via oxygen depletion, in order to mimic the hypoxic *in vivo* latency conditions. In addition, nutrient depletion models^8,9^ may mimic other conditions encountered *in vivo*, and a recently published *in vivo* model utilises hollow fibres to form what the authors describe as granulomas.^10^

The DosR/DosS/DosT regulatory system has been described in a number of *Mycobacterium* species^11–15^ and is expressed in response to hypoxia and nitric oxide stress by controlling the expression of a set of ∼48 genes termed the DosR regulon.^15^ More recently, this regulatory system has been shown to respond to carbon monoxide, which is generated in macrophages in response to *Mtb* infection^16,17^ It is thought to be important for adaptation to hypoxia, NO and CO as well as a long term survival in the host.^13,14,16–20^ The identification of the function of these genes may have allowed the elucidation of the role of this regulon in hypoxia-induced persistence, but 44 of the 48 genes encode conserved hypothetical proteins. However, 6 of these conserved hypothetical proteins are from a protein family called universal stress proteins (USPs) and we hypothesized that determining the function of these proteins would help to define non-replicating persistence, along with determining the function of an as yet poorly understood protein family.

The USP domain is a conserved domain of 140–160 amino acids and is widespread throughout the Archaea, bacteria, plants and fungi, yet it is absent in humans, making the USPs potential drug targets. With few exceptions such as *Mycoplasma* species, USPs are present in all Eubacteria and Archaea, and while some bacteria, such as *Mycobacterium leprae*, have a single USP most have multiple USPs.^21^ The domain name originates from the UspA of *Escherichia coli*, which was named due to its upregulation in response to a wide variety of stresses. Mutation of the *E. coli uspA* gene results in a survival defect under a variety of growth-arrested conditions, while overexpression induces a growth arrested state.^22–24^ *E. coli* has six *usp* genes, which have as yet undefined roles in functions ranging from oxidative stress to adhesion and motility.^25^

In the enteric bacterium *Salmonella typhimurium* a *uspA* mutant showed reduced survival following nutrient starvation and increased sensitivity to oxidative stress and it makes an important contribution to virulence.^26^ In the opportunistic pathogen *Pseudomonas aeruginosa* USPs have been shown to play a role in bacterial survival under anaerobic stationary phase; conditions thought to resemble those present in the cystic fibrosis lung colonized by *P. aeruginosa*.^27,28^ The structures of a number of bacterial USP proteins have been resolved, with ATP present in the crystal structure of MJ0577 from *Methanococcus jannaschii*,^29^ while that of UspA from *Haemophilus influenzae* had no bound ATP.^30^ This has led to the idea that the USP domain family falls into two groups, those proteins represented by the ATP binding structure of MJ0577 and those represented by the non-ATP binding structure of UspA,^21,30,31^ with the putative ATP binding USPs having a G2×G9×GS motif that forms the ATP binding site of MJ0577.

*Mtb* has 10 USPs in total and interestingly, 6 of these are DosR-regulated and their expression is also upregulated in macrophages^15,32^ leading us to hypothesize that they may be playing a role in persistence and/or intracellular survival. The USPs of *Mtb* are upregulated and overlap in their response to many different stresses, including low pH,^33^ nitric oxide,^32^ UV light and mitomycin C^34^ (Table 1). This work aimed to investigate the role of USPs from *Mtb* in the survival of this bacterium in response to a variety of stresses. We have constructed, complemented and phenotypically characterized 4 USP mutants of *Mtb*; 3 are DosR-regulated (*Rv1996, Rv2005c, Rv2028c*) and while Rv2026c is not DosR-regulated it is adjacent in the genome to DosT (Rv2027c), one of the two histidine kinases that interact with DosR and is thought to be primarily responsible for sensing hypoxia. Surprisingly we found no discernable phenotype in response to a wide variety of stresses and growth conditions, raising the possibility that USPs are functionally redundant in *Mtb.*

## 2 Materials and methods

### 2.1 Growth of *M. tuberculosis* strains

*M. tuberculosis* H37Rv cultures were grown, at 37 °C with 150 rpm shaking, in Middlebrook 7H9 medium (Difco) plus 10% oleic acid, albumin, dextrose and catalase (OADC, Difco) and 0.05% Tween 80 (Sigma). For oxygen-sufficient cultivation 50 ml cultures were grown in 250 ml Erlenmeyer flasks. The number of bacilli was estimated either using optical density (at 600 nm) or by determination of colony forming units following plating on Middlebrook 7H10 medium (Difco), plus OADC.

### 2.2 Growth of *M. tuberculosis* strains under hypoxic conditions

Oxygen starved *Mtb* cultures were grown in 125 ml flasks in a volume of 75 ml 7H9 + OADC with 150 rpm shaking, giving a headspace ratio (HSR) of 1.67 as described for the growth of *Mycobacterium smegmatis* to a hypoxic stationary phase.^35,11^ Each culture was sealed using a rubber SubaSeal septum (number 53, Sigma) to prevent air exchange and samples of culture were removed using a 19G sterile needle through the SubaSeal to prevent the introduction of oxygen into the culture. Sealed cultures grew exponentially but leveled off at an O.D. _600nm_ of ∼0.2. Preliminary experiments in which the HSR were varied inferred that with an HSR of 1.67 cultures entered stationary phase due to oxygen-starvation, as was the case with *M. smegmatis*.^35^

### 2.3 Determination of colony forming units (CFUs)

In 96-well plates, a 10-fold dilution series of the cultures were prepared in PBS + 0.05% Tween 80. Growing cultures were diluted in 100 μl, transferring 10 μl between each well, to serially dilute down to the 10^−8^ dilution. 20 μl spots of each dilution were dropped onto dry 7H10 plates in triplicate and allowed to dry before incubating at 37 °C for 2 weeks, at which point the colonies were counted.

### 2.4 Mutant construction

Two different approaches were used for constructing *usp* mutants.

(i) *sacB* counter selection method. *Mtb* H37Rv Δ*Rv2026c* and Δ*Rv2028c* mutants were generated using a *sacB* counter selection.^36^ ˜1 kb regions of DNA flanking the *Rv2026c* and *Rv2028c* genes were PCR-amplified from the genomic template. The *Rv2026c* primers were as follows: Upstream Flanking Sequence (UFS) F: tctagaatgacggttgccgtcagcggaacg and R: tctagaggctgtcgcagcagacatttcacg, both of which have a 5′ XbaI site and Downstream Flanking Sequence (DFS) F: tctagagtcgtccgtcccagctagtactgg, with a 5′ XbaI site and R: gatatcttgcgcgaggtccagggcggggtc, with a 5′ EcoRV site. The *Rv2028c* primers were: UFS F: actagaccgcgaatcatcactttgaccatg, with a 5′ SpeI site and R: gggccctttgtgtgattggttcatggcgag with a 5’ApaI site and DFS F: tctagaggtcagcagtatctgtgactgtgc with a 5′ XbaI site and R: catatgaagccggtcgaaccggcggggcgt with a 5′ NdeI site. The UFS and DFS regions were cloned either side of the hygromycin cassette in the suicide vector pSMT100 to make a marked mutant of Rv2026c and upstream of the hygromycin to make an in-frame deletion of Rv2028c^37^ and transformed into *E. coli* DH5α and plasmids from transformants screened by PCR analysis for successful constructs. Following electroporation of the constructs into *Mtb H37Rv* and plating onto 7H10 plus hygromycin (100 μg/ml), single crossovers were purified by three rounds of re-streaking onto fresh hygromycin-containing plates. Gene replacement events (double crossovers) were selected for by growing colonies in liquid 7H9 media in the presence of 5% sucrose for 1 week and plated onto 7H10 plus 5% sucrose (100 μg/ml hygromycin was also included for the marked *Rv2026c* mutation), prior to re-streaking. Confirmation of gene deletion was carried out by PCR using primers outside of the upstream and downstream flanking regions, and observing a shift in size of the PCR product for the mutants. The PCR products were cloned into pGEM-T (Promega) and sequenced to confirm the mutation.
(ii) Specialised transduction. The mycobacteriophage-based method of specialized transduction,^38^ which utilizes conditionally replicating shuttle phasmids created from pHAE159 and pYUB854, was used to create the Δ*Rv1996* and Δ*Rv2005c* mutants. Initially, upstream (UFS) and downstream (DFS) sequences flanking the *usp* gene to be mutated were amplified from *Mtb* genomic DNA using the following PCR conditions: 35 cycles of 95 °C for 30s, 55 °C for 30 s and 72 °C for 1 min. The primers used had an AflII site on the 5′ ends of UFS F, an XbaI site on the 5′ end of UFS R, HindIII on DFS F and SpeI on DFS R to allow directional insertion into the pYUBB854 vector. The primers were as follows: *Rv1996* UFS F: ggtaccgtactcgccgccaccactg, R: tctagagaaaccgcgaatactcaaat and DFS F: aagcttggcgccgatcgaatggagaaacct and R: actagtcgaagatcactg-cggcgtcaac and *Rv2005c* UFS F: cttaaggtctaccaggaggcgct, R: actagtgcgtggccttgtcgac and DFS F:tctagaccacgtcgctacatcgg and R: gacgtcgaagtggtggaa. Following PCR purification, the PCR product was ligated into the pGEM-T^amp^ vector (Promega), and then transformed into competent *E. coli* DH5α cells^39^ and plated out LB agar plus 100 μg/ml ampicillin. Following overnight incubation of isolated white colonies in LB broth plus ampicillin, PCR analysis was used as above to identify successful ligations. The cloned UFS or DFS fragment was then sub-cloned into appropriately restriction enzyme digested pYUB854*^Hyg^*(AflII*/*XbaI for UFS fragments and HindIII/SpeI *for DFS* fragments)^38^ and the recombinant pYUB854 derived plasmids transformed into *E. coli* HB101, and plasmids from transformants screened by PCR analysis for successful constructs. The temperature sensitive phage pHA159 and the pYUB854^hyg^ vector, containing both the UFS and DFS fragments for a particular *usp* gene disruption, were then digested with PacI and following gel extraction, were ligated using T4 DNA ligase to create a shuttle phasmid, which was then *in vitro* packaged using the Giga XL *in vitro* packaging mix (Stratagene) and transformed into *E. coli* HB101 cells according to the manufacturer’s instructions. Successful phasmids were identified by PacI digestion, following antibiotic selection on LB plus hygromycin (100 μg/ml) plates, purification and growth overnight in LB broth plus hygromycin (100 μg/ml). The phasmid was then transduced into *M. smegmatis* mc^2^155 at the permissive temperature of 30 °C, which allows replication and lysis, and a high titre lysate was produced as described previously.^38^ Transduction into *Mtb* was then carried out with the high titre lysate at an M.O.I. of 10:1 at the non permissive temperature of 37 °C, which results in delivery of the substrate. Cells only and phage only controls were included. Following incubation for 2–3 weeks on 7H10 plus OADC, glycerol and hygromycin, colonies were picked, grown in liquid medium (7H9 plus OADC, glycerol, Tween 80 and hygromycin) for 2 weeks and then confirmation of gene deletion was carried out as above via PCR using primers outside the upstream and downstream flanking regions and observing a shift in size for the mutants.

### 2.5 Complementation of the mutants

Complementation analyses of Rv2005c and Rv1996 were performed by cloning the full length genes carrying the predicted promoter regions of the genes into the integration proficient vector pMV306^kan^.^40,41^ Initially, the surrounding area was amplified from *Mtb* genomic DNA using gene specific primers with XbaI and HindIII sites attached to the 5′ end of the forward and reverse primers respectively to allow directional insertion into the pMV306 vector. The primer sequences were *Rv1996*F: tctagagccgacgatgacagcgt and R: tcctcgggtattacggcg and *Rv2005c* F: tctagattgaggacctaagcccgtt and R: gttggtcggtgagtccat and the PCR program and preliminary ligation stage in the pGEM-T vector was the same as used in the generation of the UFS and DFS fragments noted above. The pGEM-T clone plus the insert and pMV306^kan^ vector were then digested with the appropriate restriction enzyme, the insert and pMV306^kan^ vector gel purified using the Qiagex gel extraction kit (Qiagen) and ligated using T4 DNA ligase according to the manufacturer’s instructions. Transformation was then carried out in *E. coli* HB101 cells, plasmids were extracted using the Qiagen miniprep kit and PCR analysis was used to screen for successful constructs. The vector, plus the vector only control, was then transformed into the *Mtb* mutants using electroporation.^42^ Prior to use in experimental procedures, complementation was confirmed by PCR analysis for the targeted gene on DNA isolated from the mutant, wildtype and complemented strains. *Mtb* Δ*Rv2026c* and Δ*Rv2028c*::*hyg* were complemented with a single copy of the gene under the control of a tetracycline inducible promoter.^43^ The full length *Rv2026c* and *Rv2028c* genes were amplified by PCR and a ribosome binding site included in the forward primer. The primer sequences were Rv2026F: ggatcctttaagaaggagtatacatatgtctgctgcgacagcgaaa and R: cccgggctagctgggacggacgacgat and Rv2028F: ggatcctttaagaaggagatatacatatgaaccaatcacacaaacc and R: gcatgctcacagatactgctgaccgac. Fragments were cloned into the pMind-Lux vector using a Klenow blunted SpeI site in the vector and BamHI, replacing the LuxAB reporter present in pMind-Lux. The region containing the tetracycline repressor, tetracycline regulatable promoter and the *usp* gene was removed from this vector using KpnI and HindIII, and cloned into the single copy, integrating vector pMV361 using Klenow blunt ended DraIII and EcoRI sites, which remove the hsp60 promoter in pMV361. RT-PCR was used to determine that Rv2026c and Rv2028c could be induced from the tetracycline regulatable promoter, restoring transcription of the genes in the mutant backgrounds.

### 2.6 Screening for survival phenotypes under stress

Stress agents (Table 1) were diluted in PBS + 0.05% Tween 80 and 500 μl aliquots added to a 24-well plate. *Mtb* strains were grown to mid-exponential or stationary phase as indicated and 10 μl aliquots of culture added to each well of the plate. Typically this corresponded to between 1 and 5 × 10^7^ cells. CFU determination was carried out at 0 h and 3 days post-inoculation, unless otherwise noted.

### 2.7 Cell culture and infection model

The murine macrophage-like cell line J774A.2 and the human monocyte-derived cell line THP-1 were obtained from the American Type Culture Collection (Manassas, VA) and maintained in RPMI medium plus l-glutamine and 10% heat inactivated fetal bovine serum (h.i. FBS, Life Tech). Viable cell number was determined using a haemocytometer and trypan blue staining (Sigma). Prior to infection, the cells were seeded in 24-well plates at 5 × 10^5^ cells/ml. THP-1 cells were pre-activated with PMA at 20 ng/ml overnight. Infection was carried out at a multiplicity of infection (M.O.I.) of 10:1 by overlaying the monolayers with bacteria re-suspended in RPMI and washing 3 times with prewarmed medium after 2 h to remove any extracellular bacilli. The infected monolayers were then incubated at 37 °C with 5% CO_2_. Successful intracellular infection was confirmed by Kinyoun staining, using the TB cold stain kit (BDH) on formaldehyde-fixed infected monolayers. All treatments were carried out in triplicate and the controls included non-infected cells.

## 3 Results

### 3.1 The universal stress proteins (USPs) of *M. tuberculosis*

**Figure 1:**
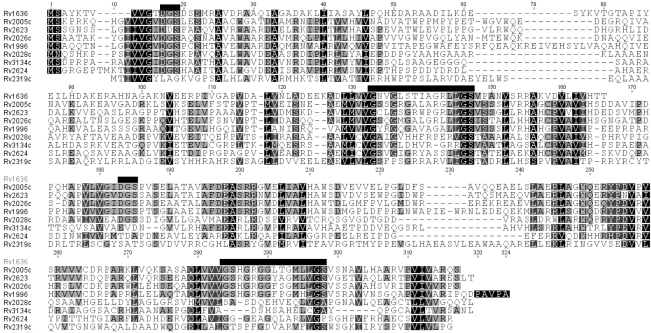
Clustal alignment of the USPs of *M. tuberculosis*. The 9 tandem domain proteins show a high degree of similarity across two key conserved motifs (DGS and G2×G9×GS, indicated above the sequence by short and long horizontal bars, respectively), but are less similar across the other regions of the proteins. Rv1636 is the only single domain USP present in *Mtb*. Rv2319c lacks some of the highly conserved residues present in all of the other USPs. Rv2005c, Rv2623, Rv2026c and Rv1996 in particular possess a high degree of similarity with each other in their second USP domain.

*M. tuberculosis* contains 10 USPA domain (PFAM PF00582)-containing proteins, all of which appear to be stand alone proteins with either single (Rv1636) or tandem domains with the exception of the KdpD (Rv1028), which contains a USP domain of unknown function as part of a sensor kinase protein. All 10 *Mtb* USPs possess a striking degree of similarity to each other within those regions identified as being conserved across all species, although this does not translate to a dramatic degree of similarity across the entire proteins. Figure 1 shows an alignment of 9 of the 10 *Mtb* USPs, (excluding KdpD). All of the tandem domain *Mtb* USPs have a G2×G9×GS conserved motif, which is part of a proposed ATP binding site^29,30^ and a conserved D (in a DGS motif) in USPA domain 1, except Rv2139 which has only a partially conserved motif (G12xGS). Four of the USPs also have these features fully conserved in UspA domain 2 (Rv2005c, Rv2623, Rv2026c and Rv1996). So all of the Mtb USPs, with the possible exception of Rv2139, fall into the putative ATP binding class of USPs.^30^

**Figure 2:**
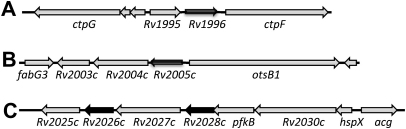
Genome context of the *M. tuberculosis usp* genes studied in this work.

The genes encoding universal stress proteins in Mtb are distributed across the chromosome and are surrounded by genes of very different or unknown function, so that it is difficult to discern a functional role based on the identification and context of the neighboring genes. The genome context of the tandem USPs studied here is shown in Figure 2. The currently available mycobacterial genome sequences were searched using a Blast search with all of the *Mtb* USP protein sequences to try to identify homologues in the related species. All of the *Mtb* USPs were determined to be conserved in *Mycobacterium bovis* and *M. microti*, with no alterations in amino acid sequence. Interestingly, *M. leprae*, the causative agent of leprosy in humans, possesses only one USP. In comparison to *Mtb*, the *M. leprae* genome has undergone extensive reductive evolution, with the loss of approximately 1700 genes, around 25% of the total genome.^44^ Blast searches of the *M. leprae* predicted proteins, using the *Mtb* USPs as query sequences, revealed that of the *Mtb* USPs, Rv1636 is the only one with a homologue in *M. leprae*. The amino acid sequences of this protein, (Ml1390), and of Rv1636 show an 89% identity with each other.

### 3.2 Construction of usp mutants

To investigate the functions of the Mtb USPs, knock-out mutants were constructed for four of the tandem domain USPs; *Rv1996, Rv2005c, Rv2026c and Rv2028c* in *Mtb H37Rv*. Three are DosR-regulated (Rv1996, Rv2005c, Rv2028c) and Rv2026c is adjacent in the genome to Rv2027c (DosT), one of two histidine kinases that interact with DosR and is thought to be responsible for hypoxia sensing. Deletion mutants of *Mtb* H37Rv lacking *Rv2026c* and *Rv2028c* were generated by a 2 step allelic exchange mutagenesis and the mutants confirmed by PCR analysis.^36,37,45^ In addition, marked deletion mutants of *Rv1996* and *Rv2005c* were constructed by a mycobacteriophage-based method of specialized transduction^46^ and the mutant constructs confirmed by PCR. Each of the mutants was complemented in single copy using pMV361 for Rv2026c::Hyg and ΔRv2028c and pMV306 for Rv1993::Hyg, and Rv2005c::Hyg.

### 3.3 Individual *usp* genes are nonessential for growth *in vitro*

**Figure 3:**
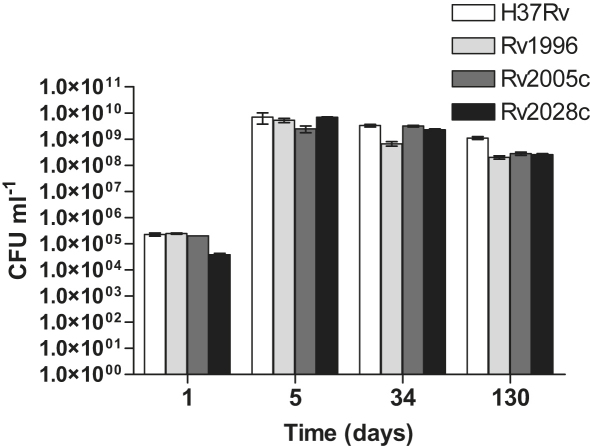
Hypoxic survival of *M. tuberculosis* H37Rv *usp* mutants. The *Mtb* tandem domain *usp* mutants were cultured to hypoxic stationary phase and survival followed for 130 days. The viability (CFUs) was measured at several time-points to determine if the mutants possess any survival defect under these conditions. Error bars represent the standard deviations of 3 independent cultures.

The *Mtb* H37Rv *usp* mutant strains were initially tested for any growth defects in broth cultures and on 7H11 plates, plus OADC and glycerol, and plus and minus Tween 80. None of the mutants tested appeared to have any discernable phenotype in liquid or solid media, with colony size and morphology appearing similar to that of the wildtype H37Rv strain (results not shown) and the growth rate and final cell density shown by each of the *usp* mutant strains was very similar to the wildtype, as were the complemented mutants. Due to the upregulation of the DosR regulon under hypoxic conditions,^15^ the fact that mutation of *dosR* compromises bacterial survival^11,14,47,48^ and that six USPs, including Rv1996, Rv2005c, and Rv2028c, are DosR-regulated, the survival of the mutants under hypoxic stationary phase conditions was investigated over a period of 130 days, but no survival defect was observed (Figure 3). Similarly, no survival differences were observed over periods of up to 80 days during normoxic stationary phase (data not shown). So none of the four *usp* genes mutated showed survival phenotypes that have been commonly associated with *usp* mutants from other bacteria.^24,26–28,49^

### 3.4 Individual *usp* genes are nonessential during *in vitro* stress challenge

**Figure 4:**
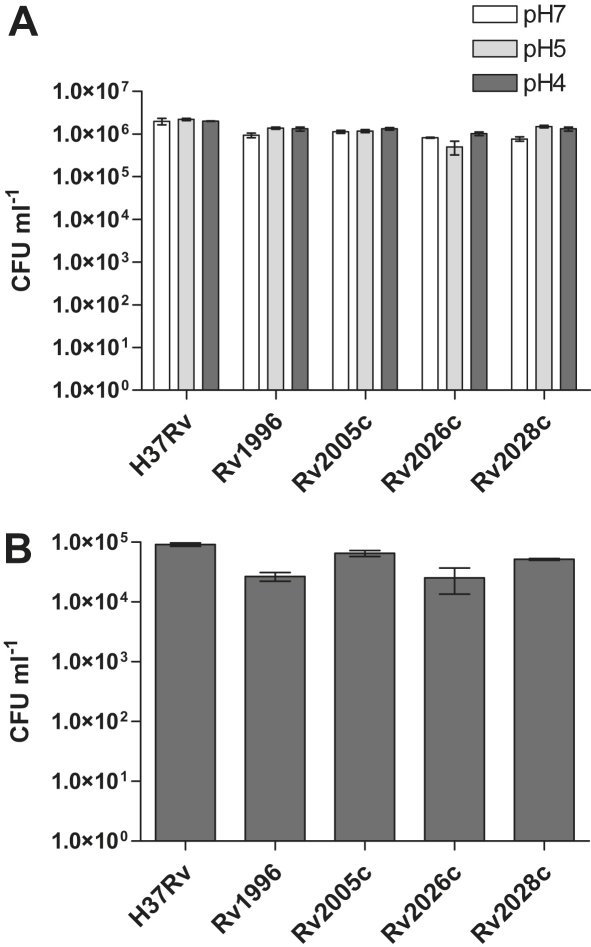
The *M. tuberculosis* usp mutants exhibited no survival defect in response to a range of stress conditions. A number of other stress conditions were also tested (see Table 1), but no survival phenotype was observed when comparing the usp mutants to the wildtype H37Rv. As examples, the results for A) low pH and B) Nitrosative stress (5 mM GSNO) are shown. Error bars represent the standard deviations of 3 independent cultures.

Mining of publically available *Mtb* microarray data identified a range of stress conditions that lead to the upregulation of one or more USPs (Table 1). Multiple studies have shown that 6 *usp* genes are upregulated by low oxygen and NO in a DosR dependent manner; 7 *usp* genes are upregulated by H_2_O_2_ and by DNA damaging agents UV light and mitomycin C, which is interesting in the context of the importance of a number of USPs from *E. coli* having a putative role in DNA protection.^50,51^ Therefore, these conditions together with others (Table 1), chosen to represent the predicted environmental conditions experienced in the disease state, were used to screen the *usp* mutants and complemented strains for survival defects compared to wildtype. The screens were performed in 24-well plates and CFU platings used to determine the survival of the cultures following the stress challenge. In all cases the survival of the *usp* mutants was found to be no different from that of the wildtype. None of the stress agents tested yielded a phenotype for any of the mutants. A representative selection of the results is shown in Figure 4.

### 3.5 Rv2026c is not involved in the expression of DosR-regulated genes

**Figure 5:**
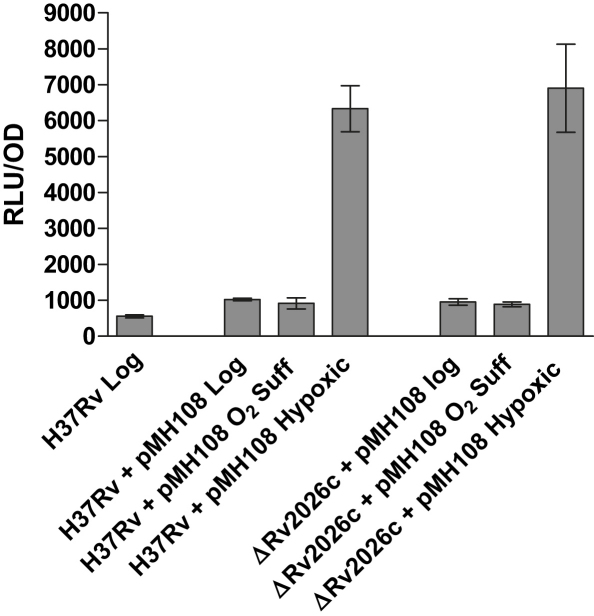
hspX promoter activity in *M. tuberculosis* H37Rv and ΔRv2026c. The plasmid pMH108 carrying the *hspX* promoter region upstream of the firefly luciferase genes was transformed into *Mtb* H37Rv and ΔRv2026c. The promoter activity was measured in cultures grown under oxygen-sufficient conditions to mid-exponential phase and stationary phase and in cultures grown to hypoxic stationary phase. Relative light units (RLU) were determined and normalised using the OD of the cultures. The *hspX* promoter was seen to be active in both the wildtype and the ΔRv2026c mutants strain. Error bars represent the standard deviations of 3 independent cultures.

Rv2026c is not a part of the DosR regulon, and its expression is not induced by hypoxia or NO, and while it is expressed during starvation in PBS (Table 1), the ΔRv2026c mutant showed a comparable survival defect to the wildtype under these conditions (data not shown). RT-PCR experiments showed that Rv2026c was transcribed during exponential phase, normoxic and hypoxic stationary phase and as expected mutation of *dosR* did not affect expression levels (not shown). Rv2026c is adjacent to *dosT* (Rv2027c) (Figure 2C), encoding the DosT histidine kinase partner of DosR, and its apparent constitutive expression led us to consider whether this USP might be involved in DosR mediated induction of genes. Both the sensor kinases DosT and DosS activate the response regulator DosR, resulting in induction of the DosR regulon, with the evidence being that DosT preferentially responds to hypoxia,^52^ and a *dosT* mutant was only able to induce DosR regulon expression to 40–45% of normal levels (Roberts et al., 2004). The possible role of Rv2026c in the DosR pathway was tested using a luciferase reporter assay with a plasmid construct containing the *hspX* promoter region upstream of firefly luciferase gene.^20^ The marked induction of *hspX* expression under hypoxic conditions occurred in wildtype H37Rv, as expected, and the ΔRv2026c mutant showed no difference in *hspX* expression indicating that Rv2026c is not involved in the expression of DosR-regulated genes (Figure 5).

### 3.6 Growth and survival of usp deletion mutant strains in macrophage models of infection

**Figure 6:**
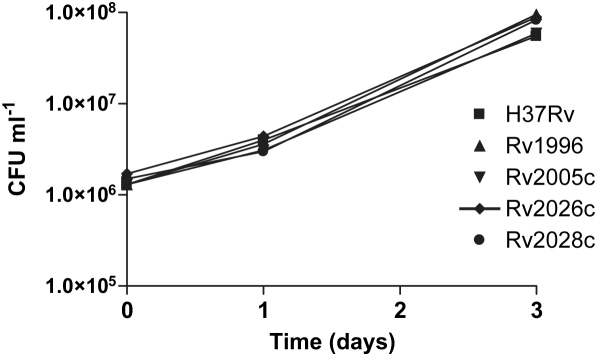
Intracellular survival of the usp mutants in the murine macrophage-like cell line J774 plus IFN-gamma. The usp mutants were tested for a survival defect in a macrophage cell line by determining CFU/ml over a 3-day time course. A similar curve was obtained for the inactivated macrophage infection.

As all of *M. tuberculosis* USPs, with the exception of Rv1636 and *kdpD*, are upregulated during macrophage infection,^32^ we determined whether any of our *usp* mutants exhibited a survival deficiency in macrophages. Initially, we looked at survival in the murine macrophage-like cell line J774 (Figure 6), and by the human monocyte-derived cell line THP-1, which was pre-activated with PMA (results not shown). No significant differences in growth or survival were noted with any of the mutants tested. This was also the case when the cells were pre-activated overnight with interferon gamma (at 10 U/ml; results not shown). Therefore, we can conclude that none of the *usp* mutants tested has a discernable intracellular survival phenotype.

## 4 Discussion

This paper reports the construction of isogenic *Mtb* mutants, with disruptions in four genes encoding for the universal stress proteins Rv1996, Rv2005c, Rv2026c and Rv2028c, and their phenotypic analysis. In bacteria there is a consistent link between USPs and survival under growth arrested conditions. Mutation of *uspA* gene of *E. coli* leads to premature death in stationary phase.^22,23^ A *S. typhimurium uspA* mutant is less virulent, more sensitive to oxidative stress and survives carbon or phosphorous starvation poorly,^26^ while work on USPs from the major periodontal pathogen *Porphyromonas gingivalis* indicates a role in anaerobic biofilm survival.^49^ In the opportunistic pathogen *P. aeruginosa*, the USP PA3309 is essential for survival during pyruvate fermentation and is expressed in anaerobic biofilms,^28^ whereas the USP PA4352 is important for survival during anaerobic stationary phase under nitrate limitation and in the presence of the uncoupler CCCP.^27^ Therefore, USPs are important for stationary phase survival of *P. aeruginosa* under exactly the types of survival conditions thought to exist in the CF lung. There are parallels with *Mtb* in which 6 USP genes are upregulated by low oxygen conditions (Table 1). We tested the survival of *Mtb usp* mutants under a variety of non-growing and other stress conditions selected in part based upon published expression data (Table 1). Our results show that individual *usp* genes encoding Rv1996, Rv2005c, Rv2026c and Rv2028c can be mutated in *Mtb* without any effect on growth or survival under normoxic or hypoxic stationary phase conditions. Mutation of the gene encoding the response regulator DosR, which regulates Rv1996, Rv2005c and Rv2028c, leads to a hypoxic survival defect in *Mtb*, as do mutations of *dosR* genes in *M. bovis* BCG and *M. smegmatis*,^11,47^ but clearly the phenotype does not result from a failure to express one of the four *usp* genes mutated here, despite the fact three are upregulated by hypoxia (Table 1). The genes encoding Rv1996, Rv2005c, Rv2026c and Rv2028c are also upregulated in activated human^53,54^ and mouse macrophages.^32^ However, in our studies, we found that there was no significant difference in intracellular growth between the wildtype and *usp* mutants in murine or THP-1 macrophage cell lines, either non-activated or activated with IFNγ. We have not tested the mutant in bone-marrow derived macrophages or in a mouse model of infection, however, so it remains to be determined if our *Mtb dosR* mutant is deficient in models more closely representing the *in vivo* situation.

While the exact function of the *Mtb* tandem domain USPs studied here still remains to be determined, we believe that these results are interesting as they suggest that the functions of the *Mtb* USPs are either partially or completely redundant. A redundant function for the USPs would result in the disruption of the single *usp* genes being compensated for by continuing expression of the remaining homologues. Our investigation of the published microarray data has indicated that the *in vitro* expression patterns of these genes do overlap, suggestive of potentially overlapping functions. However, despite the overlaps in expression, the USPs do show some evidence of differential regulation. One possibility is that the individual members or small subsets of the *Mtb* USP family serve different functions in specific phases of an infection, although they are nonessential when knocked out. A similar result to that described here has previously been reported for the *Mtb rpf* genes, which are nonessential unless knocked out in multiples of more than one.^55–58^ However, whereas the USPs of *E. coli* each seem to play a role in defense against DNA damaging agents they do not show redundancy in this role as individual mutants in *uspA*, *uspC*, *uspD* and *uspE* each show increased sensitivity to UV light but the evidence suggests that they operate and are required in the same pathway.^50,51^

The diverse genomic contexts of the Mtb *usp* genes may enable prediction of their role based upon the function of neighboring genes impossible. Biochemical characterization of these USPs will be important to elucidate their individual functions. UspA from *E. coli* is a serine threonine phosphoprotein whereas phosphorylation *in vivo* is dependent on the phosphoprotein TypA.^59,60^ MJ0577 from *M. jannaschii* had a tightly bound ATP in its crystal structure,^29^ whereas in the structure of UspA from *H. influenzae* did not have an ATP in its structure.^30^ UspG of *E. coli* is post-translationally modified during its overexpression in *E. coli* and *in vitro* experiments showed it to have intrinsic autophosphorylation and autoadenylation activity.^61^ A recent publication on another tandem domain *usp* from Mtb indicates it can also bind ATP. As this work was being written up for publication a comprehensive study of the tandem domain USP Rv2623 from Mtb was published.^62^ Rv2623 is also part of the DosR regulon and indeed is regulated by similar environmental conditions as the hypoxia-induced USPs studied here (Table 1). The ΔRv2623 mutant was unable to establish a chronic infection in mice and guinea pigs, exhibiting a hypervirulent phenotype with increased bacterial load and mortality and the overproduction of Rv2623 led to an attenuation of bacterial growth *in vitro*. Furthermore, consistent with our findings here, the Rv2623 mutant did not show any *in vitro* growth differences to the wildtype and was no more sensitive than the wildtype to a range of environmental stress conditions, including DNA damaging agents, oxidative stress and heat shock and acid shock.^62^ The lack of *in vitro* growth phenotypes for Rv2623 and Rv2026c contradicts the TRaSH screening experiments for identifying essential *Mtb* genes in which both USPs were found to be essential for *in vitro* survival (Table 1).^63^ While many of the genes identified in *Mtb* using TRaSH screenings are conserved in *M. leprae*, neither Rv2623 nor Rv2026c have homologues in *M. leprae* and, while an innovative approach, TRaSH does have limitations (see Ref.^64^). Clearly, there could be additional environments *in vitro* which we have not examined under which these proteins are necessary for survival and investigation of their role in long term survival in an appropriate animal model may also illuminate phenotypic differences.

The ability of *Mtb* to survive a range of rapidly changing conditions that other species would not be able to withstand could therefore explain why it has evolved to possess more USPs than most other species, and why these proteins have apparently redundant functions. However, the testing of the redundancy hypothesis requires the evaluation of *Mtb* strains harboring multiple disruptions of the *usp* genes, which is currently under way.

## Figures and Tables

**Table 1:** Summary of published expression studies in which data from *usp* gene expression is reported and the conditions chosen for the stress screening assays performed on *usp* mutants in this current study. The experimental conditions were chosen to represent the range of environmental onslaughts encountered by the bacillus during the disease state, from the period spent outside the host during transmission (cold shock), to the cell wall damaging surfactants in the lung (SDS) to the nitrosative and oxidation stresses experienced in the macrophage. Concentrations of stress agents were chosen through a search of the literature or through the testing of stress agents.

Stress condition	Exact condition tested in current work	USP Induction									
		Rv1636 (TB15.3)	*Rv1996*	*Rv2005c*	*Rv2026c*	*Rv2028c*	Rv2319c	Rv2623*† (TB31.7)	Rv2624c†	Rv3134c†	Rv1028
Low O_2_^13^ (2 h at 0.2% O_2_)	Survival under hypoxic and normoxic SP over 130 days (see methods section)			U		U		U	U	U	
Low O_2_^65^ (1% O_2_ in chemostat)			U	U		U		U	U	U	
Low O_2_ − P^66^ (22–26 days of anaerobic growth)				U							
DosR-regulated^15^ (*dosR* mutant versus wildtype. *m* = motif upstream)			Um	Um		U		U	U	Um	
*ΔsigC* versus wildtype in Mtb CDC1551^67^									U (MT2699)		
*ΔhspR* versus wt^68^				U							
ΔrelA_MTB_ versus wildtype during starvation in Tris-buffered saline plus Tween^69^		D (6 h)	U (4 h/6 h)								
NO/low O_2_^14^ (DETA/NO 50 μm for 40 min/0.2% O_2_ for 2 h)			U	U		U		U	U	U	
NO^70^ (100 μm NOR-3 and SPER-NO for 4 h)			U	U		U		U	U		
Palmitic acid^32^ (0.05 M for 1 h)		U									
H_2_O_2_^32^ (5 mM for 40 min)											
H_2_O_2_(4 mM for 12h) [See Gene Expression Omnibus Accession number GDS326 (http://www.ncbi.nlm.nih.gov/sites/GDSbrowser?acc=GDS326)]		U		U	U	U	U		U	U	U
UV/Mitomycin C (0.2 μg/ml Mitomycin C/variable UV exposure for up to 12 h) [See Gene Expression Omnibus Accession number GDS326 (http://www.ncbi.nlm.nih.gov/sites/GDSbrowser?acc=GDS326)]		U		U	U	U	U		U	U	U
Low pH^33^ (pH 5.5 for 15 min)											
PBS starvation 4 h^8^						D				D	U
PBS starvation 24 h^8^											U
PBS starvation 96 h^8^			D			D				D	U
Shaking versus standing cultures^71^ (7 days–promoter activity of chosen genes determined)				U				U			
Low O_2_^71^ (1.3% – promoter activity of chosen genes determined as above)				U				U			
Intra versus extracellular growth^71^ (48 h infection in J774A.2 macrophages versus tissue culture medium)				U				U			
IFN- γ activated MΦ^32^ (Murine bone-marrow derived macrophages infected at an M.O.I. of 2–5. 4, 24 and 48 h post-infection)			U	U	U	U	U (48 h only)	U	U	U	
NOS2−/− MΦ^32^ (Murine bone-marrow derived macrophages from NOS2−/− mice infected at an M.O.I. of 2–5. 24 h post-infection)					U	U	U				
Murine infection^72^ (Balb/c and SCID mice infected intranasally with 10^3^ CFU. 7, 14 and 21 days post-infection)			U at 7/14/21 days in Balb/c and at 21 days in SCID	U (14 days/Balb/c)				U (21 days/Balb/c)			
*In vitro* survival^63^(TraSH essentiality screen)					E		E	E			E§
	SDS 0.05 and 0.1%–3 days										
	55 °C heat shock, 5 h and 72 h										
	Osmotic shock, 2.5 M NaCl (72 h)										
	Exposure to Isoniazid 0.1, 1 μgml^−1^ (72 h)										
	Cold shock 4 °C for up to 72 h										
	Ethanol 5, 10% (72 h)										

All regulatory data from published microarray studies, except for proteomic studies (P). U = Up-regulated, D = down-regulated, MΦ = macrophage, E = essential for optimal growth *in vitro*, SP = stationary phase.

∗ Expressed in the murine lung, concurrent with the onset of Th1 immunity.^72^

† In transposon screen for intracellular survival, mutants in Rv2623 and Rv2624c grew better in un-activated macrophages and were attenuated in pre-activated macrophages, whilst an Rv3134c mutant grew best in post-activated cells.^73^

§ Essential *in vivo*^74^
